# Enhancing the Analytical and Sensory Quality of Warm-Climate Tempranillo Wines Through Co-Inoculation with *Lachancea thermotolerans* and *Metschnikowia pulcherrima*

**DOI:** 10.3390/foods15071249

**Published:** 2026-04-06

**Authors:** Fernando Sánchez-Suárez, Simone Nesi, Giovanni Caruso, Nieves López de Lerma, Rafael A. Peinado

**Affiliations:** 1 Agricultural Chemistry, Soil Science and Microbiology Department, Campus of Rabanales, University of Córdoba, N-IV Road, Km 396, 14014 Córdoba, Spain; g62sasuf@uco.es (F.S.-S.); b92lolem@uco.es (N.L.d.L.); 2 Department of Agriculture, Food and Environment, University of Pisa, Via del Borghetto 80, 56124 Pisa, Italy; simone.nesi@phd.unipi.it (S.N.); giovanni.caruso@unipi.it (G.C.)

**Keywords:** non-*Saccharomyces*, *Lachancea thermotolerans*, *Metschnikowia pulcherrima*, co-inoculation, aroma compounds, aromatic series, Tempranillo, warm-climate wines

## Abstract

Using non-*Saccharomyces* yeasts is a promising way to modulate wine composition, particularly in warm regions where high sugar levels result in wines with high alcohol content and low acidity. This study examined the impact of various inoculation methods involving *Lachancea thermotolerans* and *Metschnikowia pulcherrima* on the chemical and aromatic profiles of Tempranillo wines. Five treatments were tested: a control with *Saccharomyces cerevisiae*; two sequential inoculations, with each non-*Saccharomyces* species followed by *S. cerevisiae*; and two co-inoculations of both species at different ratios, followed by *S. cerevisiae*. *L. thermotolerans* was found to significantly increase lactic acid levels, resulting in higher acidity and lower pH, particularly at higher doses. Wines inoculated with *M. pulcherrima* exhibited the highest levels of fruity esters, whereas *L. thermotolerans* increased higher alcohols, acetaldehyde and ethyl lactate, but reduced ester content. Co-inoculations produced intermediate or synergistic effects, depending on the proportions of the yeast species. Aromatic analysis indicated that treatments involving *M. pulcherrima* were associated with higher contributions to the fruity, floral and waxy aromatic series, while *L. thermotolerans* increases creamy series. Multivariate analysis clearly differentiated wines according to the dominant species. Overall, the co-inoculation of both yeasts is an effective method of modulating volatile composition and aroma-related parameters in warm-climate red wines.

## 1. Introduction

In recent decades, climate change has emerged as a major challenge for the global wine industry. Rising global temperatures cause grapes to ripen earlier, resulting in higher sugar content and significantly reduced acidity, primarily due to the degradation of malic acid [1,2,3,4]. Consequently, wines produced in warm regions tend to have a higher ethanol content and higher pH, as well as lower acidity. This has a negative impact on their sensory balance, freshness, and microbiological stability [5,6]. Furthermore, these conditions may alter the synthesis of varietal aromatic compounds, reducing wine aromatic complexity [7,8]. These changes have driven the development of new oenological strategies aimed at restoring the chemical and sensory balance of wines without compromising their typicity.

Traditionally, alcoholic fermentation has been dominated by *Saccharomyces cerevisiae* yeast due to its high tolerance of ethanol and efficient fermentation [9]. However, in recent years, considerable interest has emerged in non-*Saccharomyces* yeasts, which form part of the natural grape and must microbiota [10,11]. These species possess distinctive metabolic traits that can positively influence wine composition.

*Lachancea thermotolerans*, a non-*Saccharomyces* yeast, has received special attention due to its ability to produce lactic acid from sugars during fermentation [12,13,14,15]. This bioacidification capacity naturally increases the acidity and reduces the pH of the wine, thereby improving its freshness, microbiological stability and sensory balance [16,17,18]. Furthermore, this species can influence the aromatic profile of wine by producing compounds such as higher alcohols and major esters, such as ethyl lactate, which contribute to its sensory complexity [19,20]. However, other studies have reported reductions in aromatic potential, particularly the reduction of minor ethyl esters [17,21].

Conversely, *Metschnikowia pulcherrima* has demonstrated significant oenological potential thanks to its capacity to enhance the aromatic profile of wine. This yeast can produce high levels of esters associated with fruity and floral aromas, which contribute significantly to the aromatic complexity of wine [22,23,24]. It also exhibits enzymatic activity, including β-glucosidase, which releases varietal aromatic compounds from non-volatile precursors found in grapes [24,25,26]. When co-inoculated with *Saccharomyces cerevisiae*, *Metschnikowia pulcherrima* helps to produce wines with differentiated volatile composition and aroma-related profiles, without compromising fermentation efficiency [24,25,27,28].

Overall, using non-*Saccharomyces* yeasts is a promising biotechnological strategy for improving wine quality and mitigating the negative effects of climate change. Specifically, species such as *Lachancea thermotolerans* and *Metschnikowia pulcherrima* can naturally increase acidity and improve aromatic complexity, resulting in more balanced wines that are better suited to current climatic conditions.

However, the effects of interactions between yeasts, particularly with regard to the aromatic profile or inoculation dose, and the use of fermentations involving two or more non-Saccharomyces yeasts remain insufficiently characterised. Only a few studies are available on the subject, including some by the authors of this paper [20,29,30]. This study specifically evaluates the influence of different sequential and co-inoculation strategies involving *Lachancea thermotolerans* and *Metschnikowia pulcherrima* at various ratios on the chemical composition and aromatic profile of Tempranillo wines, which typically have a high pH and low acidity in warm climates.

## 2. Materials and Methods

### 2.1. Harvest and Vinifications

Tempranillo grapes from a vineyard owned by Viñas de Alange, S.A. in Alange, Badajoz, Spain (38°40′21″ N 6°16′20″ W) were used. The grapes were manually harvested in 15 kg boxes. They were then mechanically destemmed and crushed and divided into 15 fermenters, each containing 15 kg of the harvested grapes.

At harvest time, the grapes were in optimal condition, with a sugar content of 245 g/L, a pH level of 3.44 and total acidity of 5.08 g/L, expressed as tartaric acid.

All vinifications were performed in triplicate as independent biological replicates. In all cases, commercial yeasts from Lallemand Inc. (Madrid, Spain) were used: *Saccharomyces cerevisiae* (Velluto Evolution^®^), *Lachancea thermotolerans* (Level2 Laktia^®^) and *Metschnikowia pulcherrima* (Level2 Flavia^®^). This allows a direct link to be established between this research and the wine sector. The following inoculations were performed: (1) control with *S. cerevisiae*; (2) sequential inoculation of *L. thermotolerans* and *S. cerevisiae* after 48 h; (3) sequential inoculation of *M. pulcherrima* and *S. cerevisiae* after 48 h; (4) co-inoculation of *M. pulcherrima* and *L. thermotolerans* at a ratio of 1:2, followed by sequential inoculation of *S. cerevisiae* after 48 h; and (5) co-inoculation of *M. pulcherrima* and *L. thermotolerans* at a ratio of 2:1, followed by sequential inoculation of *S. cerevisiae* after 48 h. All inoculations were performed at 25 g/hL, except for the double doses at 50 g/hL, which were used to increase the presence of the second yeast, in line with the methods of other authors [31].

Malolactic fermentation took place alongside alcoholic fermentation. The malolactic bacterium *Lactiplantibacillus plantarum* (ML Prime^®^, Lallemand Bio, S.L.) was inoculated 24 h after the initial inoculation of *S. cerevisiae*.

Alcoholic fermentation took place at a controlled temperature of 21 ± 1 °C. The cap was kept moist by punching down the grapes every 24 h to promote the extraction of phenolic compounds from the skins. Fermentation progress was monitored by taking daily density measurements until values below 995 g/L were reached. Once fermentation was complete, the grape mass was pressed using a pneumatic press.

The wine was then clarified using a mixture of vegetable protein (Proveget 100^®^, Agrovin S.A., Alcázar de San Juan, Spain) and bentonite (Bengel^®^, Agrovin S.A.), at doses of 15 and 25 g/hL, respectively.

### 2.2. Enological General Parameters

The general parameters of the wine (pH, titratable acidity, volatile acidity, ethanol content and colour index) were determined in accordance with official procedures [32]. Additionally, the malic and lactic acid content was determined using enzymatic methods and reflectometry with Reflectoquant™ equipment (Merck^®^, Darmstadt, Germany).

### 2.3. Analysis of Volatile Compounds

Volatile compounds detected in must and wine were classified into two categories based on their concentration levels: major volatile compounds (≥10 mg/L) and minor volatile compounds (<10 mg/L). All analyses were conducted in triplicate using three independent biological replicates.

#### 2.3.1. Major Volatile Compounds

The major volatile compounds and polyols were quantified using an Agilent Technologies HP 6890 Series II gas chromatograph (Palo Alto, CA, USA), which was fitted with a 50 m long, 0.25 mm internal diameter, 0.4 µm film thickness CP-WAX 57 CB capillary column and equipped with a flame ionisation detector (FID). The analytical procedure followed the methodology described by Peinado et al. [33].

For each analysis, 0.5 µL of the prepared wine sample was injected. The samples consisted of 10 mL of wine that had been spiked with 1 mL of 4-methyl-2-pentanol (1024 mg/L) as the internal standard. Prior to injection, tartaric acid was removed by precipitation using 0.2 g of calcium carbonate, followed by centrifugation at 300× *g*.

The chromatographic parameters were set as follows: a split ratio of 30:1 and FID detection. The oven temperature programme started at 50 °C and was held for 15 min. It then increased at a rate of 4 °C per minute up to 190 °C, where it was held for a further 35 min. The injector and detector temperatures were set to 270 °C and 300 °C, respectively. Helium was used as the carrier gas with an initial flow rate of 0.7 mL/min, which was maintained for 16 min. This was then increased by 0.2 mL/min to achieve a final flow rate of 1.1 mL/min, which was sustained for a further 52 min. Compound identification and quantification were carried out by analysing authentic standards under identical chromatographic conditions.

#### 2.3.2. Minor Volatile Compounds

These compounds were determined in two consecutive steps, both of which had previously been described in detail by López de Lerma et al. [34].

In the first step, volatile compounds were extracted using stir bar sorptive extraction (SBSE) technique using PDMS. Stir bars (10 mm in length with a 0.5 mm PDMS film thickness; Gerstel GmbH, Mülheim an der Ruhr, Germany) were placed in vials containing 10 mL of a 1:10 diluted sample, along with 0.1 mL of hexyl butyrate (0.4116 mg/L) as the internal standard. The samples were stirred at 1500 rpm for 100 min to allow the adsorption of the volatile compounds onto the stir bars. After extraction, the stir bars were removed and transferred into thermal desorption tubes for subsequent chromatographic analysis.

In the second step, the extracted volatile compounds were analysed by gas chromatography coupled with mass spectrometry (GC–MS) using a Gerstel TDS 2 thermal desorption unit. The stir bars were placed inside the desorption tubes and heated to 280 °C to release the adsorbed compounds. These compounds were then adsorbed in a CIS 4 programmed temperature vaporisation (PTV) inlet system, which was maintained at 25 °C and equipped with a Tenax adsorption trap. The CIS was then heated to transfer the concentrated compounds into the GC–MS system, which contained an Agilent-HP-5MS capillary column (length: 60 m; internal diameter: 0.25 mm; film thickness: 0.25 µm). The mass spectrometer operated in electron impact mode at 70 eV, scanning a mass range of 35–550 Da.

Compound identification was achieved by injecting analytical standards under the same chromatographic conditions as the samples, and by comparing the resulting retention times and mass spectra with those contained in the NIST and Wiley spectral library. Quantification was performed using calibration curves as was described by Palenzuela et al. [35].

#### 2.3.3. Calculation of Aromatic Series

Odour activity values (OAVs) were calculated by dividing the concentration of each volatile compound by its corresponding odour perception threshold (Supplementary Material Table S1 [34,36,37,38,39,40,41,42,43,44,45,46,47,48,49,50,51,52,53,54,55,56,57]).

Aromatic series were defined by grouping volatile compounds that shared similar sensory descriptors. The overall value of each aromatic series was determined by summing the OAVs of the compounds belonging to that series. Nine aromatic categories were established: chemical, green, citrus, creamy, floral, fruity, green fruit, honey and waxy. Depending on their sensory properties, individual volatile compounds could be included in one or more aromatic series.

### 2.4. Statistical Analysis

An ANOVA statistical analysis was performed to determine whether the observed differences were statistically significant. When significant differences were identified, a Tukey post hoc analysis (*p* < 0.05) was conducted to categorise them into homogeneous groups. All analyses were performed using IBM SPSS Statistics 25 (Armonk, NY, USA).

A cluster heatmap was also created using open-source Python programming language (version 3.9.7) within the Anaconda Jupyter Project environment (Anaconda Inc., Austin, TX, USA).

## 3. Results and Discussion

### 3.1. Enological General Parameters

Table 1 shows the general parameters analysed in the different wines. The ability of *L. thermotolerans* yeast to produce lactic acid from sugars has been well documented [12,14,58,59]. This influences other parameters, such as an increase in titratable acidity and a decrease in pH (Table 1). The increase in lactic acid observed during the co-inoculation of *L. thermotolerans* and *M. pulcherrima* appears primarily associated with the higher inoculation dose of *L. thermotolerans* rather than a true synergistic interaction. Although synergistic effects have been reported in mixed fermentations [20,29,30], in this study lactic acid production increased only when *L. thermotolerans* was inoculated at 50 g/hL. When the dose of *L. thermotolerans* was 25 g/hL and *M. pulcherrima* 50 g/hL, lactic acid production was even lower than in the sequential Lt + Sc treatment. These findings are consistent with Vicente et al. [31], who demonstrated that lactic acid production increases when the inoculation rate of *L. thermotolerans* exceeds that of *S. cerevisiae*.

Volatile acidity increases slightly when *L. thermotolerans* is involved in fermentation. Similar results have been obtained by other researchers, who have explained this by reference to the rebalancing of the reducing power in the cell cytoplasm resulting from lactic acid production [17,30]. In all cases, however, the volatile acidity values can be considered adequate for a high-quality red wine.

Regarding the parameters related to phenolic composition, wines produced with *L. thermotolerans* intervention have higher colour intensity values, particularly when inoculated with *M. pulcherrima*. This effect may be attributed to enhanced stability of anthocyanins under lower pH conditions, together with possible improvements in copigmentation phenomena during early fermentation stages [60]. The same occurs with total polyphenol index.

### 3.2. Volatile Compounds

A total of 58 volatile compounds were identified and grouped into the following chemical families: alcohols, esters, aldehydes, ketones, lactones, terpenes and norisoprenoids.

Table 2 shows the concentrations of the identified volatile compounds in wines produced using different inoculation protocols. The presence of non-*Saccharomyces* yeasts was found to cause significant changes in practically all the analysed chemical families, confirming their ability to modulate the aromatic profile of wine, as previously described by various authors [22,61,62,63].

In the alcohol group, treatments containing a high proportion of *L. thermotolerans* (Lt + Sc and Mp + Lt 1:2) had the highest levels of major alcohols, showing significant differences compared to the control and Mp + Sc treatments. This increase was primarily due to elevated levels of propanol and isobutanol, consistent with previous reports describing the influence of *L. thermotolerans* on fermentative metabolism and cellular redox balance [17]. The higher production of these alcohols may reflect an enhanced flux through the Ehrlich pathway, driven by alterations in amino acid catabolism and redox rebalancing associated with lactic acid formation. Regarding minor alcohols, the highest total value was achieved by the Mp + Lt (1:2) treatment, primarily due to a significant increase in hexanol. Conversely, the Mp + Sc treatment exhibited the lowest levels of minor alcohols, particularly hexanol and octanol. These compounds are associated with green and herbaceous aromas and originate from the lipoxygenase pathway of grape fatty acids [64]. Given that the reduction of C6 aldehydes to their corresponding alcohols is generally almost complete during alcoholic fermentation, the higher hexanol levels observed in treatments involving *L. thermotolerans* are unlikely to be related to species-specific reductive activity. Rather, they may reflect differences in precursor extraction from grape tissues or indirect effects of fermentation dynamics on C6 compound retention. Conversely, 2-phenylethanol exhibits significantly lower values in treatments involving *L. thermotolerans*, with the lowest value observed in the Mp + Lt (2:1) treatment. This behaviour can be explained by differences in nitrogen metabolism of each yeast species [65].

The most pronounced differences between the treatments were observed in the minor esters, which are key compounds associated with fruity aroma descriptors [64]. The Mp + Sc treatment had the highest total concentration, significantly exceeding the other treatments. In contrast, Lt + Sc and Mp + Lt (1:2) showed marked decreases in minor ester content, consistent with previous studies describing a reduction in acetate and ethyl ester synthesis in the presence of *L. thermotolerans* [17,26,30,31,66]. This effect may be associated with alterations in acetyl-CoA availability and cellular redox balance linked to lactic acid production, which can limit ester biosynthesis. The reduction was particularly evident for isoamyl acetate, ethyl octanoate, ethyl decanoate, hexyl acetate and 2-phenylethyl acetate, all of which are major contributors to fruity aroma descriptors. However, certain esters such as ethyl propanoate and ethyl isobutanoate reached significantly higher values in the Mp + Lt (1:2) treatment, indicating a selective modulation of ester formation depending on the inoculation strategy. Among the major esters, ethyl lactate showed the most pronounced increase in treatments involving *L. thermotolerans*, reaching its highest concentration in Mp + Lt (1:2). This behaviour is directly related to the increased availability of lactic acid produced by *L. thermotolerans*, which enhances its chemical esterification with ethanol during fermentation and early post-fermentation stages [9]. Nevertheless, co-inoculation of both non-*Saccharomyces* yeasts, particularly when *M. pulcherrima* predominated, partially restored the ester content reduced by *L. thermotolerans*, with potential implications for aroma-related characteristics (see Section 3.2.1).

In the aldehyde group, acetaldehyde exhibited significantly higher levels in the *L. thermotolerans* treatments (Lt + Sc and Mp + Lt 1:2). This increase is consistent with the need to re-establish intracellular redox equilibrium during lactate formation, as acetaldehyde accumulation may compensate for NADH/NAD^+^ imbalances generated by increased lactate dehydrogenase activity [15,17]. In contrast, while minor aldehydes displayed variable behaviour across treatments, octanal was the most markedly affected compound, showing notably lower concentrations in wines involving *L. thermotolerans*. As octanal is associated with citrus notes [64], its substantial reduction may influence aroma-related characteristics. Regarding ketones, the Mp + Lt (1:2) treatment had significantly higher acetoin content than the others, while the Lt + Sc treatment showed increased levels of 3-heptanone and acetophenone.

Lactones exhibited more modest variations, though a notable increase in butyrolactone was observed in the Mp + Lt (2:1) treatment. Conversely, γ-nonalactone exhibited higher concentrations in the control and Mp + Sc treatments. This behaviour suggests that the dominant species in fermentation has a specific effect on these compounds associated with lactic and ripe fruit notes [64].

Finally, significant increases were observed in the group of terpenes and norisoprenoids in treatments involving non-*Saccharomyces* yeasts, particularly *L. thermotolerans*. The maximum value of β-citronellol was reached in Lt + Sc, while nerolidol and methyl dihydrojasmonate increased significantly in the co-inoculations, particularly in Mp + Lt (1:2) and Mp + Lt (2:1). These results are consistent with those reported by other authors, who attribute this effect to enhanced β-glucosidase activity and improved acid-catalysed hydrolysis of glycosylated terpene precursors under lower pH conditions [27,63,64,67].

Overall, the results in Table 2 confirm that *M. pulcherrima* promotes the synthesis of fruit esters and certain terpenes, while *L. thermotolerans* increases the production of alcohols, acetaldehyde, ethyl lactate and carbonyl compounds while simultaneously reducing the content of certain minor esters. Co-inoculations showed intermediate or partially synergistic profiles. Mp + Lt (1:2) stood out due to its high levels of minor alcohols, acetoin and ethyl lactate. Mp + Lt (2:1) showed partial recovery of esters and increased levels of terpene compounds.

#### 3.2.1. Aromatic Series

To facilitate interpretation of the results, the volatile compounds were grouped into different aromatic series by summing their odour activity values. This approach has been widely used in wine aroma studies [17,35,56,68,69]. Nine aromatic series were established in total, providing an overview of the changes induced by the different inoculation protocols (Table 3).

The most significant differences between the treatments were observed in the Fruity, Green Fruit and Waxy series. The wine made with *M. pulcherrima* (Mp + Sc) had the highest values of these three series and showed significant differences compared to the other treatments and the control. In contrast, the Lt + Sc wine had the lowest values, confirming the reducing effect of *L. thermotolerans* on fruit esters as described by other authors [17,31,66]. These series mainly consist of esters, the most notable of which are the ethyl esters of hexanoic, octanoic, butanoic and propanoic acids, as well as isoamyl acetate. The latter has the highest odour activity value in the Fruity series and contributes aromatic notes reminiscent of banana [64]. Co-inoculations with non-*Saccharomyces* yeasts exhibited intermediate behaviour. Notably, Mp + Lt (2:1) exhibited significantly higher values than Lt + Sc and Mp + Lt (1:2) in the aforementioned series. These results indicate that increasing the relative proportion of *M. pulcherrima* mitigates the suppressive effect of *L. thermotolerans* on ester biosynthesis.

Similar trends were observed in the Green and Honey series, with the highest values found in Mp + Sc, the control group, and Mp + Lt (2:1). Phenylacetaldehyde was the main contributor in both series, together with hexanal in the Green series and 2-phenylethyl acetate in the Honey series. Phenylacetaldehyde can be formed through oxidation of 2-phenylethanol and via Strecker degradation of phenylalanine under oxidative conditions [64,70]. In turn, the concentration of this alcohol is closely related to the nitrogen metabolism of the yeast species and the secretion of enzymes [71,72].

Significantly higher values were observed in the control wine of the Citrus series, followed by Mp + Sc, while the lowest values were observed in Lt + Sc. This behaviour is primarily driven by the higher concentrations of octanal, nonanal and decanal, which are key contributors to citrus-related aroma perception [64].

In contrast, the highest values in the Creamy series were found in the treatments with the highest presence of *L. thermotolerans*, particularly in Mp + Lt (1:2) and Lt + Sc. This behaviour is directly related to the higher concentration of ethyl lactate in these wines, formed by the esterification of ethanol with lactic acid produced by *L. thermotolerans* [9,73].

In the Floral series, the Mp + Sc treatment had the highest value, which was significantly higher than that of the other wines. This is due to a higher content of 2-phenylethanol and 2-phenylethanol acetate, which are compounds that are characteristic of floral aromas and are closely related to yeast nitrogen metabolism through the Ehrlich pathway. Several studies have described the high capacity of certain *M. pulcherrima* strains to produce these compounds, achieving concentrations much higher than those obtained with *S. cerevisiae* [74].

#### 3.2.2. Multivariate Analysis of the Aromatic Series

**Figure 1:**
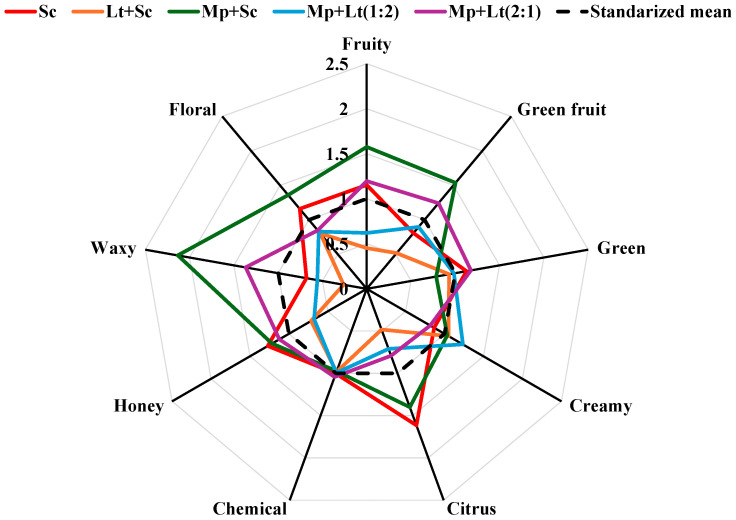
Star plots of the aromatic series. Sc: *S. cerevisiae*; Lt + Sc: sequential inoculation of *L. thermotolerans* and *S. cerevisiae* after 48 h; Mp + Sc: sequential inoculation of *M. pulcherrima* and *S. cerevisiae* after 48 h; Mp:Lt (1:2): co-inoculation of *M. pulcherrima* and *L. thermotolerans* at a ratio of 1:2, followed by sequential inoculation of *S. cerevisiae* after 48 h; Mp:Lt (2:1): co-inoculation of *M. pulcherrima* and *L. thermotolerans* at a ratio of 2:1, followed by sequential inoculation of *S. cerevisiae* after 48 h.

Multivariate analysis in the form of standardised radial graphs integrating the nine aromatic series (Figure 1) provided an overview of the volatile composition of the wines, enabling the simultaneous visualisation of differences between various inoculation protocols. Each polygon reflects the relative contribution of the main aromatic series—fruity, green fruit, green, creamy, citrus, chemical, honey, waxy and floral—to the total volatile profile. Standardising the axes enabled direct comparison between samples, revealing the impact of inoculation type and the proportion of non-*Saccharomyces* yeasts on the aromatic series. In the graph, the values are expressed in standardised form, with the broken black line corresponding to the mean value (equal to 1 after standardisation). Thus, series above this value indicate an above-average aromatic contribution, while those below reflect a lower relative intensity.

The analysis clearly distinguishes two types of behaviour among the wines studied. On the one hand, the wines in the Lt + Sc and Mp + Lt (1:2) series have a profile characterised by below-average values in most aromatic categories, particularly Fruity, Green Fruit, Waxy, Floral and Citrus. In contrast, these treatments show above-average values in the Creamy series, indicating an aromatic profile dominated by compounds associated with lactic and creamy notes. This behaviour is consistent with the greater presence of *L. thermotolerans* in these treatments, which reduces fruit esters and increases ethyl lactate.

In contrast, the control, Mp + Sc and Mp + Lt (2:1) wines have more similar aromatic profiles, with above-average values in the fruity, green fruit and waxy series, and in some cases in floral and honey. This pattern indicates a greater contribution of compounds associated with fruit esters and aromatic alcohols, resulting in a volatile profile associated with fruit and floral aroma descriptors. Within this group, the Mp + Sc treatment exhibits the highest values in the fruit series, whereas the Mp + Lt (2:1) treatment exhibits an intermediate profile, suggesting a compensatory effect between the two non-*Saccharomyces* yeasts.

The radial representation provides a distinctive aromatic fingerprint, allowing clear differentiation of wines according to dominant metabolic patterns. Treatments dominated by *L. thermotolerans* are characterised by a profile with lower contribution of fruit-related aromatic series and greater creamy notes, while treatments with a higher proportion of *M. pulcherrima* are associated with higher contribution of fruit- and floral-associated compounds.

### 3.3. Cluster Heatmap Analysis

**Figure 2:**
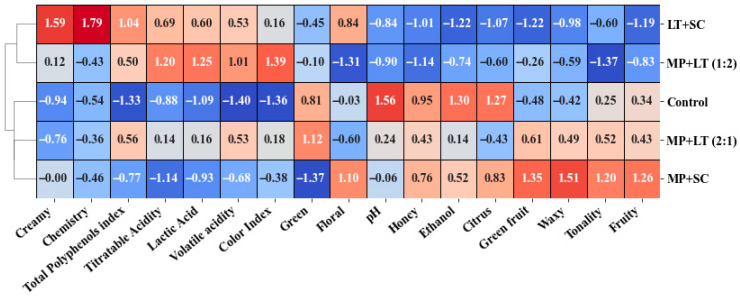
Cluster heatmap analysis obtained for the produced wines. Values above 0 indicate a positive correlation (red), whereas values below 0 indicate a negative correlation (blue). Control: *S. cerevisiae*; Lt + Sc: sequential inoculation of *L. thermotolerans* and *S. cerevisiae* after 48 h; Mp + Sc: sequential inoculation of *M. pulcherrima* and *S. cerevisiae* after 48 h; Mp:Lt (1:2): co-inoculation of *M. pulcherrima* and *L. thermotolerans* at a ratio of 1:2, followed by sequential inoculation of *S. cerevisiae* after 48 h; Mp:Lt (2:1): co-inoculation of *M. pulcherrima* and *L. thermotolerans* at a ratio of 2:1, followed by sequential inoculation of *S. cerevisiae* after 48 h.

The cluster analysis was represented by a heatmap based on standardised values to simultaneously visualise the relationships between the different wines and the analysed variables (Figure 2). In this representation, positive values indicate direct correlations between variables and are shown in shades of red, the intensity of which increases with the value of the correlation. Conversely, negative values reflect inverse correlations and are represented by shades of blue, which become more intense as the absolute value of the negative correlation increases.

The analysis identified two main groups of wines that were clearly differentiated in terms of their chemical composition and aromatic profile. The first group included wines made with a higher proportion of *L. thermotolerans* (Lt + Sc and Mp + Lt 1:2). These wines showed positive correlations with variables associated with higher acidity, such as titratable acidity, lactic acid and volatile acidity. This translates into intense red tones in the relevant boxes on the heatmap. At the same time, they showed negative correlations with variables such as pH and ethanol content (blue tones), confirming the acidifying effect and slight reduction in alcohol content associated with this yeast.

From an aromatic perspective, these wines exhibited negative correlations with most of the aromatic series dominated by esters, particularly the Fruity, Green Fruit and Waxy series. This suggests that these compounds have a lower relative contribution in the presence of *L. thermotolerans*. Conversely, positive correlations were observed with the Creamy series, reflecting an increase in ethyl lactate and other compounds associated with lactic and creamy notes. These results confirm the role of *L. thermotolerans* in modulating the aromatic profile towards less fruity and more creamy descriptors.

The second group included the control wine and treatments where *M. pulcherrima* was the only non-*Saccharomyces* yeast inoculated (Mp + Sc), or where its proportion was greater in co-inoculation (Mp + Lt 2:1).

These wines showed positive correlations with the aromatic series associated with esters, particularly the Fruity, Green fruit, and Waxy series. This is reflected by intense red tones in these variables. This behaviour indicates greater production or preservation of fruit esters, resulting in a volatile profile characterized by higher contributions of ester-related compounds.

By contrast, these treatments exhibited negative correlations with acidity-related variables, such as lactic acid and titratable acidity. This confirms the differential effect of *M. pulcherrima* compared to *L. thermotolerans*. Notably, the Mp + Sc wine exhibited a correlation pattern closely resembling the control for acidity- and pH-related variables, while Mp + Lt (2:1) exhibited intermediate values, suggesting a combined effect of both yeasts.

Analysis of the colour intensities in the heat map therefore enables the variables with the greatest discriminatory power to be identified. The most intense negative correlations (shown in blue) were concentrated between the fruit series and the acidity-related variables in treatments dominated by *L. thermotolerans*, suggesting a metabolic trade-off between acidification and ester synthesis under the studied fermentation conditions. Conversely, the most intense positive correlations (red) were observed between the Fruity, Green Fruit and Waxy series in treatments with a high presence of *M. pulcherrima*, demonstrating the direct relationship between this yeast and the formation of fruity compounds.

Overall, the heat map confirms that the dominant species and their relative proportions in co-inoculations are the most influential factors in determining the chemical and aromatic profile of wine. Treatments with a predominance of *L. thermotolerans* are associated with more acidic and lower contribution of ester-related aromatic series, whereas treatments with a greater presence of *M. pulcherrima* exhibit higher relative abundance of ester-related compounds and are therefore closer to the control. These findings corroborate the results of the volatile compound and aromatic series analyses, reinforcing the potential of targeted co-inoculation strategies as a precision tool for aroma modulation.

## 4. Conclusions

The results of this study confirm the significant impact of non-*Saccharomyces* yeasts on the chemical and aromatic composition of Tempranillo wines from warm climates. *Lachancea thermotolerans* was found to have a strong acidifying effect, increasing the concentration of lactic acid, total acidity and colour intensity while reducing the level of ethanol. It also promoted the formation of higher alcohols, acetaldehyde and ethyl lactate, resulting in wines with a volatile profile associated with creamy-related aromatic series and lower levels of fruity esters.

By contrast, *Metschnikowia pulcherrima* increased the production of fruity and floral esters, producing wines with higher scores in the fruity, green fruit, waxy and floral aromatic series. Wines produced with this yeast exhibited acidity profiles similar to the control but with enhanced ester-related complexity.

Co-inoculation strategies produced intermediate or synergistic effects, depending on the relative inoculation ratio. Treatment with a higher proportion of *L. thermotolerans* resulted in the greatest acidification and the highest levels of ethyl lactate and minor alcohols. In contrast, treatment dominated by *M. pulcherrima* partially restored ester content, enhancing terpene-related compounds. Multivariate analyses confirmed the clear separation between wines dominated by each non-*Saccharomyces* species, highlighting the decisive influence of inoculation strategy on the final aromatic profile.

Overall, the combined use of *L. thermotolerans* and *M. pulcherrima* represents a robust biotechnological strategy to mitigate the impact of climate change on warm-climate red wines. By fine-tuning inoculation ratios, winemakers can strategically modulate acidity, redox balance and ester biosynthesis, enabling the production of wines with modified acidity balance and differentiated volatile profiles.

## Figures and Tables

**Table 1:** Enological general parameters determined in the produced wines.

		Control	Lt + Sc	Mp + Sc	Mp:Lt (1:2)	Mp:Lt (2:1)
pH		3.71 ± 0.04 a	3.58 ± 0.03 b	3.62 ± 0.04 ab	3.57 ± 0.03 b	3.64 ± 0.04 ab
Titratable acidity	(g/L TH_2_)	4.65 ± 0.07 d	6.56 ± 0.08 b	4.3 ± 0.3 d	7.18 ± 0.04 a	5.9 ± 0.02 c
Ethanol	(% *v*/*v*)	14.5 ± 0.1 a	13.6 ± 0.2 c	14.2 ± 0.1 a	13.8 ± 0.2 bc	14.1 ± 0.2 ab
Volatile acidity	(g/L AcH)	0.3 ± 0.03 c	0.38 ± 0.02 ab	0.33 ± 0.03 bc	0.4 ± 0.02 a	0.38 ± 0.02 ab
Lactic acid	(g/L)	0.89 ± 0.04 d	2.08 ± 0.06 b	1.00 ± 0.09 d	2.5 ± 0.1 a	1.77 ± 0.08 c
Color Index		15.3 ± 0.4 c	17.8 ± 0.9 b	16.9 ± 0.4 bc	20 ± 1 a	17.8 ± 0.2 b
Tonality		33 ± 3 a	32 ± 1 a	34 ± 1 a	31 ± 3 a	33 ± 1 a
Total Polyphenols index		23.4 ± 0.4 c	28 ± 2 a	24.4 ± 0.7 bc	26.6 ± 0.6 ab	27 ± 1 ab

Data are expressed as mean ± SD. TH_2_: Tartaric acid; AcH: Acetic acid; Control: *S. cerevisiae*; Lt + Sc: sequential inoculation of *L. thermotolerans* and *S. cerevisiae* after 48 h; Mp + Sc: sequential inoculation of *M. pulcherrima* and *S. cerevisiae* after 48 h; Mp:Lt (1:2): co-inoculation of *M. pulcherrima* and *L. thermotolerans* at a ratio of 1:2, followed by sequential inoculation of *S. cerevisiae* after 48 h; Mp:Lt (2:1): co-inoculation of *M. pulcherrima* and *L. thermotolerans* at a ratio of 2:1, followed by sequential inoculation of *S. cerevisiae* after 48 h. Different letters indicate significant differences at 95% confidence level.

**Table 2:** Volatile aroma compounds quantified in the produced wines.

	Control	Lt + Sc	Mp + Sc	Mp:Lt (1:2)	Mp:Lt (2:1)
**Major alcohols (mg/L)**	**572 ± 9 c**	**589 ± 3 b**	**562 ± 3 d**	**599.8 ± 0.6 a**	**571 ± 2 c**
Methanol	72 ± 5 a	57 ± 8 a	71 ± 9 a	63 ± 5 a	58 ± 1 a
Propanol	45 ± 5 b	65 ± 5 a	50 ± 2 b	64 ± 4 a	54 ± 2 ab
Isobutanol	59 ± 4 c	78 ± 4 a	63 ± 3 bc	78 ± 4 a	71 ± 4 ab
2-Methylbutanol	40 ± 2 a	40 ± 2 a	41.1 ± 0.9 a	42 ± 3 a	43.7 ± 0.3 a
3-Methylbutanol	302 ± 28 a	309 ± 8 a	288 ± 7 a	312 ± 3 a	311 ± 7 a
2-Phenylethanol	53 ± 1 a	40.2 ± 0.9 b	50 ± 3 a	42 ± 4 ab	33.2 ± 0.5 c
**Minor alcohols (µg/L)**	**3256 ± 306 ab**	**3427 ± 258 a**	**2593 ± 236 c**	**3629 ± 150 a**	**2668 ± 95 bc**
Hexanol	3071 ± 304 ab	3304 ± 250 a	2478 ± 237 c	3509 ± 137 a	2599 ± 94 bc
2-Ethyl-1-hexanol	23 ± 2 a	23 ± 2 a	23 ± 2 a	24 ± 2 a	22.1 ± 0.4 a
Octanol	151 ± 3 a	87 ± 4 b	80 ± 5 b	81 ± 8 b	37 ± 3 c
Decanol	5.9 ± 0.6 a	5.4 ± 0.6 a	5.7 ± 0.5 a	5.4 ± 0.4 a	4.1 ± 0.2 b
Dodecanol	2.2 ± 0.2 a	2.4 ± 0.2 a	2.3 ± 0.2 a	1.27 ± 0.07 b	1.5 ± 0.1 b
Farnesol	2.7 ± 0.1 d	6.1 ± 0.5 b	4.5 ± 0.3 c	9.6 ± 0.9 a	4.5 ± 0.2 c
**Major esters (mg/L)**	**127 ± 4 b**	**119.9 ± 0.5 c**	**118 ± 1 c**	**133 ± 1 a**	**117 ± 1 c**
Ethyl acetate	86 ± 9 a	71 ± 1 b	76 ± 3 ab	67 ± 3 b	77 ± 3 ab
Ethyl lactate	32 ± 2 c	41 ± 1 b	33 ± 1 c	58 ± 1 a	30.3 ± 0.3 c
Diethyl succinate	8.7 ± 0.4 bc	7.84 ± 0.09 c	8.8 ± 0.4 b	9.1 ± 0.3 b	10.2 ± 0.1 a
**Minor esters (µg/L)**	**8014 ± 362 b**	**3014 ± 88 d**	**9244 ± 334 a**	**3583 ± 78 d**	**6722 ± 472 c**
Ethyl propanoate	39.8 ± 0.4 d	62 ± 2 b	30 ± 3 e	71 ± 1 a	53 ± 2 c
Ethyl isobutanoate	15 ± 1 d	31 ± 2 b	15 ± 1 d	37 ± 2 a	23 ± 2 c
Ethyl butanoate	179 ± 14 a	102 ± 1 c	152 ± 10 b	121 ± 2 c	145 ± 9 b
Butyl acetate	0.9 ± 0.05 a	0.69 ± 0.06 cd	0.55 ± 0.06 d	0.85 ± 0.04 ab	0.72 ± 0.06 bc
Isoamyl acetate	5554 ± 285 a	1842 ± 95 c	5588 ± 147 a	2096 ± 70 c	4316 ± 440 b
Ethyl hexanoate	367 ± 16 c	234 ± 9 d	700 ± 38 a	408 ± 12 c	565 ± 13 b
Hexyl acetate	148 ± 5 a	16.6 ± 0.8 d	108 ± 4 b	21.2 ± 0.5 d	49 ± 1 c
Ethyl heptanoate	0.25 ± 0.03 d	0.35 ± 0.01 c	0.52 ± 0.01 b	0.48 ± 0.04 b	0.75 ± 0.01 a
Ethyl benzoate	0.33 ± 0.04 a	0.22 ± 0.01 b	0.26 ± 0.02 b	0.22 ± 0.02 b	0.23 ± 0.02 b
Ethyl octanoate	126 ± 10 c	27 ± 3 e	470 ± 15 a	94 ± 7 d	305 ± 9 b
Ethyl phenylacetate	163 ± 12 a	1.9 ± 0.2 d	49 ± 4 b	1.1 ± 0.1 d	24.9 ± 0.9 c
2-Phenylethanol acetate	1182 ± 24 b	577 ± 36 d	1784 ± 142 a	553 ± 36 d	936 ± 12 c
Ethyl decanoate	146 ± 3 c	71 ± 3 e	280 ± 3 a	120 ± 5 d	235 ± 7 b
2-Phenylethyl butanoate	24 ± 1 a	2.2 ± 0.2 b	2.9 ± 0.2 b	1.6 ± 0.1 b	2.1 ± 0.2 b
Phenethyl hexanoate	1.64 ± 0.06 a	0.68 ± 0.04 b	0.4 ± 0.02 d	0.53 ± 0.01 c	0.38 ± 0.01 d
Ethyl tetradecanoate	28 ± 3 a	16 ± 2 b	25 ± 3 a	13 ± 1 b	16 ± 1 b
Phenethyl benzoate	1.8 ± 0.2 a	1.75 ± 0.06 a	1.8 ± 0.1 a	1.28 ± 0.04 b	1.3 ± 0.08 b
Ethyl hexadecanoate	37 ± 5 b	27 ± 3 c	37 ± 4 b	41 ± 4 ab	50 ± 3 a
**Major aldehydes (mg/L)**	**55 ± 5 b**	**79 ± 4 a**	**59 ± 3 b**	**84 ± 3 a**	**51.4 ± 0.6 b**
Acetaldehyde	55 ± 5 b	79 ± 4 a	59 ± 3 b	84 ± 3 a	51.4 ± 0.6 b
**Minor aldehydes (µg/L)**	**133 ± 4 a**	**47 ± 2 e**	**104 ± 1 b**	**64 ± 4 d**	**78 ± 2 c**
Benzaldehyde	2.4 ± 0.3 a	2.1 ± 0.2 a	1.3 ± 0.1 b	2.4 ± 0.1 a	2.5 ± 0.2 a
Hexanal	12 ± 0.3 ab	11.1 ± 0.6 b	10.9 ± 0.5 b	14 ± 1 a	13 ± 1 ab
Heptanal	1.4 ± 0.2 ab	1.2 ± 0.1 bc	1.4 ± 0.1 ab	1.62 ± 0.09 a	0.98 ± 0.09 c
Octanal	80 ± 3 a	1.6 ± 0.1 e	59 ± 3 b	12 ± 1 d	27 ± 0.4 c
Nonanal	9 ± 0.8 abc	8 ± 1 bc	12 ± 1 a	11 ± 1 ab	8 ± 0.2 c
Decanal	8.4 ± 0.6 a	8.4 ± 0.8 a	9.5 ± 0.8 a	9 ± 1 a	4.9 ± 0.5 b
Phenylacetaldehyde	20 ± 2 a	14 ± 2 b	11 ± 1 b	13 ± 1 b	21 ± 1 a
**Major Ketones (mg/L)**	**14 ± 1 c**	**16.2 ± 0.8 b**	**13.9 ± 0.5 cd**	**20 ± 1 a**	**12.5 ± 0.5 d**
Acetoin	14 ± 1 c	16.2 ± 0.8 b	13.9 ± 0.5 cd	20 ± 1 a	12.5 ± 0.5 d
**Minor Ketones (µg/L)**	**4.5 ± 0.3 c**	**6.8 ± 0.5 a**	**5.4 ± 0.3 bc**	**5.6 ± 0.2 b**	**5.4 ± 0.3 bc**
Benzophenone	1.66 ± 0.09 a	0.9 ± 0.1 b	0.71 ± 0.08 bc	0.62 ± 0.07 c	0.5 ± 0.03 c
3-Heptanone	2.2 ± 0.2 c	3.8 ± 0.4 a	2.9 ± 0.2 bc	3.2 ± 0.3 ab	2.6 ± 0.2b c
Acetophenone	0.58 ± 0.06 c	2.1 ± 0.2 ab	1.8 ± 0.2 b	1.8 ± 0.2 b	2.3 ± 0.2 a
**Lactones (µg/L)**	**7452 ± 667 a**	**1228 ± 130 b**	**1785 ± 200 b**	**1945 ± 101 b**	**1877 ± 233 b**
Butyrolactone	7438 ± 666 a	1195 ± 132 b	1759 ± 203 b	1916 ± 99 b	1856 ± 232 b
γ-Nonalactone	13 ± 1 bc	10 ± 1 c	21 ± 2 a	15 ± 2 b	15 ± 1 b
γ-Decalactone	0.66 ± 0.05 d	23 ± 2 a	4 ± 0.3 c	14 ± 1 b	5.6 ± 0.5 c
**Terpenes & Norisoprenoids (µg/L)**	**47 ± 2 c**	**80 ± 4 a**	**64 ± 3 b**	**68 ± 3 b**	**76 ± 2 a**
Limonene	15.5 ± 0.7 c	24.6 ± 0.5 b	24 ± 3 b	27 ± 1 b	35 ± 2 a
β-Citronellol	23 ± 1 c	45 ± 5 a	29 ± 1 bc	35 ± 3 b	36 ± 3 b
E-Citral	2.1 ± 0.2 a	2.3 ± 0.3 a	1 ± 0.1 b	0.89 ± 0.09 b	0.26 ± 0.03 c
Z-Nerolidol	0.09 ± 0.01 c	0.18 ± 0.01 c	0.17 ± 0 c	1.8 ± 0.2 a	0.64 ± 0.03 b
E-Geranyl acetone	2 ± 0.2 b	4.2 ± 0.3 a	4.8 ± 0.5 a	0.44 ± 0.03 c	0.63 ± 0.03 c
Z-Geranyl acetone	2.5 ± 0.1 a	2.5 ± 0.1 a	2.7 ± 0.2 a	1.6 ± 0.1 b	1.54 ± 0.05 b
E-Methyldihydrojasmonate	2 ± 0.2 a	1.2 ± 0.1 b	2.07 ± 0.08 a	0.91 ± 0.02 b	1.2 ± 0.1 c

Data are expressed as mean ± SD. Control: *S. cerevisiae*; Lt + Sc: sequential inoculation of *L. thermotolerans* and *S. cerevisiae* after 48 h; Mp + Sc: sequential inoculation of *M. pulcherrima* and *S. cerevisiae* after 48 h; Mp:Lt (1:2): co-inoculation of *M. pulcherrima* and *L. thermotolerans* at a ratio of 1:2, followed by sequential inoculation of *S. cerevisiae* after 48 h; Mp:Lt (2:1) co-inoculation of *M. pulcherrima* and *L. thermotolerans* at a ratio of 2:1, followed by sequential inoculation of *S. cerevisiae* after 48 h. Different letters indicate significant differences at 95% confidence level.

**Table 3:** Aromatic series obtained for the produced wines.

	Control	Lt + Sc	Mp + Sc	Mp:Lt (1:2)	Mp:Lt (2:1)
**Fruity**	253 ± 13 b	99.2 ± 2.5 d	345 ± 9 a	136 ± 5 c	262.1 ± 18 b
**Green fruit**	27 ± 1 c	17 ± 1 d	50 ± 3 a	29 ± 1 c	41 ± 1 b
**Green**	8.6 ± 0.6 ab	7 ± 1 cd	6.0 ± 0.2 d	7.5 ± 0.5 bc	9.0 ± 0.2 a
**Creamy**	0.98 ± 0.04 c	1.3 ± 0.1 ab	1.17 ± 0.06 b	1.4 ± 0.1 a	1.0 ± 0.1 c
**Citrus**	43.8 ± 1.3 a	13 ± 1 d	38 ± 0.2 b	19.3 ± 1.2 c	21.0 ± 0.3 c
**Chemistry**	28 ± 2 a	27.0 ± 0.1 a	27.2 ± 0.7 a	27.4 ± 0.4 a	29.0 ± 0.7 a
**Honey**	10.4 ± 0.5 a	6 ± 1 b	10 ± 0.8 a	5.5 ± 0.4 b	9.0 ± 0.5 a
**Waxy**	33 ± 2 c	13 ± 1 e	103 ± 3 a	27 ± 1 d	66 ± 2 b
**Floral**	11.5 ± 0.1 b	8.1 ± 0 c	13.5 ± 0.9 a	7.8 ± 0.5 c	8.4 ± 0.1 c

Data are expressed as mean ± SD. Control: *S. cerevisiae*; Lt + Sc: sequential inoculation of *L. thermotolerans* and *S. cerevisiae* after 48 h; Mp + Sc: sequential inoculation of *M. pulcherrima* and *S. cerevisiae* after 48 h; Mp:Lt (1:2): co-inoculation of *M. pulcherrima* and *L. thermotolerans* at a ratio of 1:2, followed by sequential inoculation of *S. cerevisiae* after 48 h; Mp:Lt (2:1): co-inoculation of *M. pulcherrima* and *L. thermotolerans* at a ratio of 2:1, followed by sequential inoculation of *S. cerevisiae* after 48 h. Different letters indicate significant differences at 95% confidence level.

## Data Availability

The original contributions presented in this study are included in the article/Supplementary Materials. Further inquiries can be directed to the corresponding author.

## Supplementary Materials

The following supporting information can be downloaded at: https://www.mdpi.com/article/10.3390/foods15071249/s1, Table S1: Odour descriptor, odour threshold and aroma series assigned to the volatile compounds identified in the wines analysed.

## Abbreviations

The following abbreviations are used in this manuscript:

GC-FID	Gas chromatography–flame ionization detector
GC-MS	Gas chromatography–mass spectrometry
OAV	Odour activity value
TDS	Thermal desorption unit
