# Supplementary material for: Enhancing the Analytical and Sensory Quality of Warm-Climate Tempranillo Wines Through Co-Inoculation with Lachancea thermotolerans and Metschnikowia pulcherrima
Source: Foods. 2026 Apr 6;15(7):1249. doi: 10.3390/foods15071249 (PMC13074045; doi:10.3390/foods15071249)
Supplement: Supplementary file 1 [file foods-15-01249-s001.zip › foods-4197350-supplementary.pdf]

Table S1. *Odor descriptor, odor threshold and aroma series assigned to the volatile compounds identified in the wines analyzed.*

Compound	OPT	Descriptors	Serie	Reference
**Alcohols**				
**Mayor Alcohols**				
Methanol	668000	Chemical, medicinal, fruity	5	(Ogawa et al., 2022)
Propanol	830000	Ripe fruit, fusel alcohol	5	(Ogawa et al., 2022)
Isobutanol	40000	Nail polish, bitter	5	(Ogawa et al., 2022)
Isoamyl alcohols^{+}	30000	Burnt, alcohol	5	(Ogawa et al., 2022)
2-phenylethanol	10000	Rose, honey, lilac	9	(Ogawa et al., 2022)
**Minor Alcohols**				
Hexanol	8000	Green, grass, oily	3	(López de Lerma et al., 2018)
2-ethyl-1-hexanol	8000	Citrus, fresh	5	(Y.-S. Zhang et al., 2019)
Octanol	800	Waxy, green, citrus, aldehydic and floral	8	(Peinado et al., 2006)
Decanol	400	aldehydic waxy green	8	(Qu et al., 2024)
Dodecanol	1000	waxy, earthy, soapy, aldehydic	8	(Li et al., 2008)
Farnesol	20	floral juicy	9	(Muñoz-Castells et al., 2024)
**Esters**				
**Mayor Esters**				
Ethyl acetate	7500	Fruity, glue	5	(Guth, 1997)
Ethyl lactate	150000	Fruity, byttery	4	(Peinado et al., 2004)
Diethyl succinate	100000	Overripe melon	1	(Ogawa et al., 2022)
**Minor Esters**				
Ethyl propanoate	10	Sweet, fruity, grape	1	(Leffingwell & Associates, n.d.)
Ethyl isobutanoate	15	Sweet, etherial and fruity	1	(López de Lerma et al., 2018)
Ethyl butanoate	20	Fruity, floral	1	(Guth, 1997)
Butyl acetate	66	Sweet, ripe, banana, ethereal	1,6	(Takeoka et al., 1996)
Isoamyl acetate	30	sweet fruity banana	1	(Guth, 1997)

Compound	OPT	Descriptors	Serie	Reference
Ethyl hexanoate	14	sweet fruity pineapple green banana	1,2	(Ferreira et al., 2000)
Hexyl acetate	70	fruity green apple banana sweet	1,2	(Swiegers et al., 2005)
Ethyl heptanoate	2,2	fruity pineapple	1	(López de Lerma et al., 2018)
Ethyl benzoate	60	Minty, Fruity	1	(Buttery et al., 1988)
Ethyl octanoate	5	Banana, pineapple	1,8	(López de Lerma et al., 2018)
Ethyl phenylacetate	250	Rose, floral	7,9	*
2-Phenylethyl acetate	250	floral-rose and gardenia	7,9	(Guth, 1997)
Ethyl decanoate	200	Sweet, fruity, caramel, grape	1,8	(Gómez-Míguez et al., 2007)
2-Phenylethyl butanoate	250	Floral, yeast	9	*
Phenethyl hexanoate	250	Fruity-green, fresh pineapple-like	8,9	*
Ethyl tetradecanoate	4000	Tropical fruit	8	(Moreno, 2005)
Phenethyl benzoate	250	Rose, honey, balsamic	2,9	*
Ethyl hexadecanoate	2000	Waxy, fruity nuances	8	(Moreno, 2005)
**Aldehydes**				
**Mayor Aldehydes**				
Acetaldehyde	110000	Pungent, ripe apple	1,5	(Moreno, 2005)
**Minor Aldehydes**				
Benzaldehyde	350	Bitter almond, smoked	1	(Buttery et al., 1988)
Hexanal	5	Herbaceous, green apple	3	(Buttery et al., 1988)
Heptanal	3	Herbal, coriander	3	(Buttery et al., 1988)
Octanal	2,5	Citrus, green, fresh, waxy	5	(López de Lerma et al., 2018)
Nonanal	2,5	Citrus	5	(López de Lerma et al., 2018)
Decanal	1,25	Citrus	5,8	(López de Lerma et al., 2018)
Phenylacetaldehyde	4	green sweet honey	3,7	(Buttery et al., 1971)
**Ketones**				
**Mayor ketones**				

Compound	OPT	Descriptors	Serie	Reference
Acetoin	150000	Yogurt, butterscotch	4	(S. Zhang et al., 2015)
**Minor ketones**				
Benzophenone	65	Balsamic, rose, herbal, geraniun	9	(Leffingwell & Associates, n.d.)
3-Heptanone	7,5	Green, ketonic	3	(Burdock & Fenaroli, 2010)
Acetophenone	65	Mimosa, Acacia	9	(Leffingwell & Associates, n.d.)
**Lactones**				
Butyrolactone				
γ -Nonalactone	30	Coconut, creamy	1,4	(Ferreira et al., 2000)
γ -Decalactone	77.7	Peach, milky	1,4	(Gottmann et al., 2023)
**Terpenes & Norisoprenoids**				
Limonene	10	citrus orange fresh sweet	5,6	(López de Lerma et al., 2018)
β -Citronellol	40	Floral, Rose, citrus	9	(Leffingwell & Associates, n.d.)
E-Citral	32	Citrus, lemon	5	(Martínez-García et al., 2021)
Z-Nerolidol	700	Floral, green	9	(Martín-García et al., 2023)
E-Geranyl acetone	60	Magnolia, rose	9	(Zhu et al., 2019)
Z-Geranyl acetone	60	Magnolia, rose	9	(Zhu et al., 2019)
E-Methyldihydrojasmonate	70	Floral, oily, jasmin	9	(Martín-García et al., 2023)

1 Fruity; 2 GreenFruit; 3 Green; 4 Creamy; 5 Citrus; 6 Chemistry; 7 Honey; 8 Waxy; 9 Floral. .$^{+}$: 2-Methylbutanol + 3-Methylbutanol *Expressed as 2-phenethyl acetate; ** Expressed as Benzaldehyde; *** Expressed as Ethyl decanoate.
